# Supplementary material for: Oxidative Damages on the Alzheimer’s Related-Aβ Peptide Alters Its Ability to Assemble
Source: Antioxidants (Basel). 2023 Feb 13;12(2):472. doi: 10.3390/antiox12020472 (PMC9951946; doi:10.3390/antiox12020472)
Supplement: Supplementary file 1 [file antioxidants-12-00472-s001.zip › antioxidants-2157793-supplementary.pdf]

Oxidative Damages on the Alzheimer's Related-Aβ Peptide Alters Its Ability to Assemble

Clémence Cheignon †, Fabrice Collin ‡, Laurent Sabater and Christelle Hureau *

LCC-CNRS, Université de Toulouse, CNRS, 31077 Toulouse, France; ccheignon@unistra.fr (C.C.);
* Correspondence: christelle.hureau@lcc-toulouse.fr
† Current addresses: Equipe de Synthèse Pour l'Analyse, SynPAUniversité de Strasbourg, CNRS, IPHC UMR 7178, 67037 Strasbourg, France.
‡ Current addresses: UMR 5623 IMRCP, CNRS, 31000 Toulouse, France.

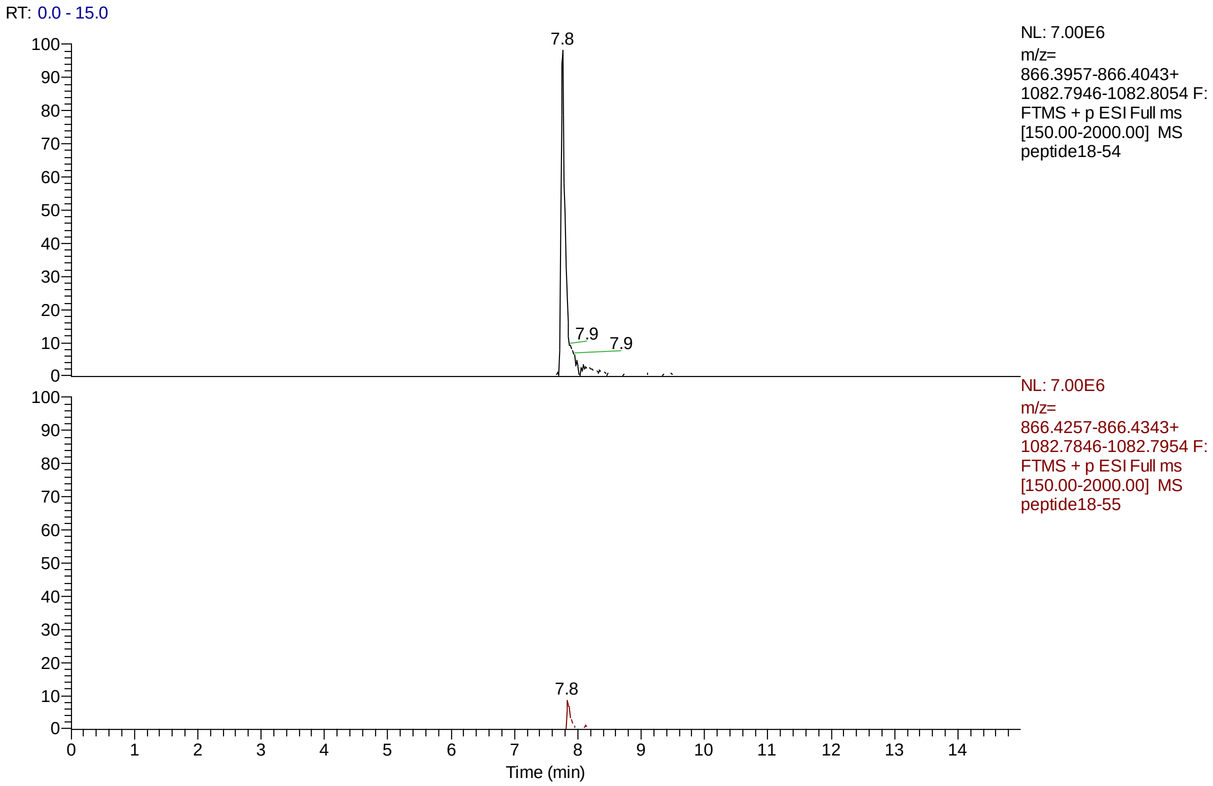

<u>Supporting Information for experiment 1.</u> Remaining non-oxidized Aβ_{1-40} – trace chromatogramms of ions at m/z 1082.7949 and 866.4375 corresponding to the $[M+4H]^{4+}$ and $[M+5H]^{5+}$ ions of Aβ_{1-40}, for aqueous solution of control and oxidized ^{ox}Aβ_{1-40}; mass accuracy was set at 5 ppm.

Characterization of $A\beta_{1-40}$ **oxidation.**

After oxidation, the solution was filtered using Amicon 3 kDa centrifugal device (Millipore) by centrifugation for 15 min at 13500 rpm, then washed and centrifuged twice with 200 µL ammonium bicarbonate (100 mM, pH 8). The concentrated sample (approx. 50 µL) was recovered and transferred to an Eppendorf ProteinLoBind 1.5 mL vial. Trypsin (proteomics grade, from Sigma, 0.05 µg/µL in formic acid 0.1%) was added to obtain a Aβ40/trypsin ratio of 20/1 (w/w) and digestion was carried out at 37°C for 3h in a Thermomixer (Eppendorf), 10 s mixing at 750 rpm every min.

After proteolytic digestion, sequencing of oxidized $A\beta_{1-40}$ was performed by High Performance Liquid Chromatography / Mass Spectrometry (LC-MS) analysis on an ion-trap mass spectrometer (LCQ DECA XP Max, ThermoScientific), equipped with an electrospray ionization source, and coupled to an Ultimate 3000 LC System (Dionex, Voisins-le-Bretonneux, France). Samples (10 µL) at 60 µM of $^{ox}A\beta_{1-40}$ were injected onto the column (Acclaim 120 C18, 50 × 3 mm, 3 µm, ThermoScientific), at room temperature. The gradient elution was carried out with formic acid 0.1% (mobile phase A) and acetonitrile/water (80/20 v/v) formic acid 0.1% (mobile phase B) at a flow-rate of 0.5 $mL.min^{-1}$. The mobile phase gradient was programmed with the following time course: 5% mobile phase B at 0 min, held 3 minutes, linear increase to 55% B at 8 min, linear increase to 100% of B at 9 min, held 2 min, linear decrease to 5% B at 12 min and held 3 min. The mass spectrometer was used as a detector, working in the full scan positive mode between m/z = 150 and 2000 followed by data dependent scans of the two first most intense ions, with dynamic exclusion enabled. Isolation width was set at m/z = 1 a and collision energy at 28% (units as given by the manufacturer), using wideband activation. The generated tandem MS data was searched using the SEQUEST algorithm against the $A\beta_{1-40}$ peptide sequence (BioWorks software, ThermoScientific). Dynamic modifications were specified according to the expected mass shift due to the $A\beta_{1-40}$ peptide oxidation. The same operating conditions than for LC-MS (column and mobile phase gradient) were used to carry out high resolution mass spectrometry (LC/HRMS) experiments, by using a LTQ-Orbitrap XL mass spectrometer (ThermoFisher Scientific, Les Ulis, France) coupled to an Ultimate 3000 LC System (Dionex, Voisins-le-Bretonneux, France). The Orbitrap cell was operated in the full-scan mode at a resolution power of 60 000. The mass spectrometer was used as a detector, working in the full scan positive mode between m/z = 150 and 1200.

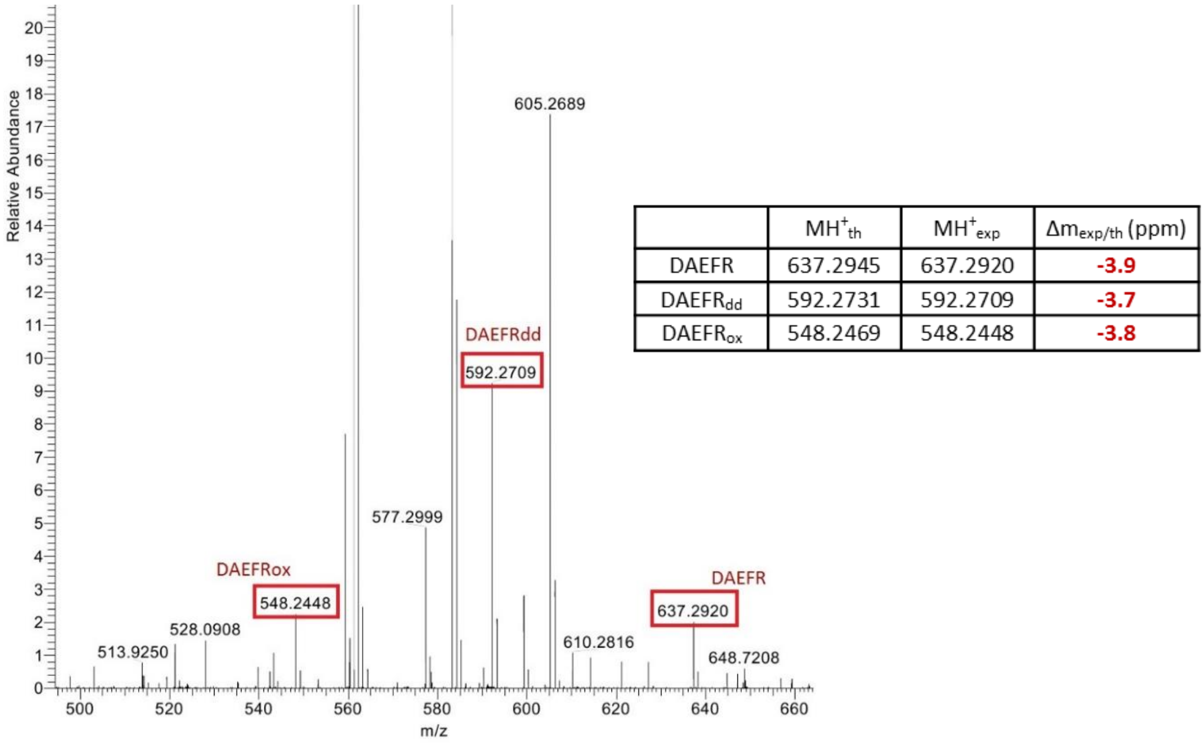

	MH^+_{th}	MH^+_{exp}	$\Delta m_{exp/th}$ (ppm)
DAEFR	637.2945	637.2920	**-3.9**
$DAEFR_{dd}$	592.2731	592.2709	**-3.7**
$DAEFR_{ox}$	548.2469	548.2448	**-3.8**

Figure S1. Full mass spectrum of DAEFR and its oxidation products obtained by high-resolution mass spectrometry (LTQ-Orbitrap, Thermoscientific) working at 60 000 resolution power, for $A\beta_{1-40}$ submitted to oxidation using the Cu-Aβ/ascorbate/dioxygen system. $DAEFR_{dd}$ = decarboxylation/deamination of Asp1; $DAEFR_{ox}$ = oxidative cleavage of Asp1-Ala2. Calculated monoisotopic masses are indicated along with experimental ones and mass difference (in ppm) between experimental and calculated masses.

DTA for scans: 104-107 Precursor ion: 676.94 Charge: 2 Activation type: CID Mass type: mono
Mod's: (DAEFRHSGYVQK* -45.02150) (DAEFRHSGYVQK# -89.05000) (DAEFRHSGYVQK@ +15.99490)

	AA	A	B	B*	Bo	C	Y	Y*	Yo	Z	
1	H		138.07				-				11
2	D		253.09				1215.54				10
3	S		340.13				1100.51				9
4	G		397.15				1013.48				8
5	Y		560.21				956.46				7
6	E		689.25				793.40				6
7	V		788.32				664.35				5
8	H@		941.37				565.28				4
9	H		1078.43				412.23				3
10	Q		1206.49				275.17				2
11	K		-				147.11				1

z=1 z=2

DTA for scans: 104-107 Precursor ion: 676.94 Charge: 2 Activation type: CID Mass type: mono
Mod's: (DAEFRHSGYVQK* -45.02150) (DAEFRHSGYVQK# -89.05000) (DAEFRHSGYVQK@ +15.99490)

	AA	A	B	B*	Bo	C	Y	Y*	Yo	Z	
1	H		69.54				-				11
2	D		127.05				608.27				10
3	S		170.57				550.76				9
4	G		199.08				507.24				8
5	Y		280.61				478.73				7
6	E		345.13				397.20				6
7	V		394.66				332.68				5
8	H@		471.19				283.15				4
9	H		539.72				206.62				3
10	Q		603.75				138.09				2
11	K		-				74.06				1

z=1 z=2

#104-107 RT:4.81-4.87 NL: 4.51E5

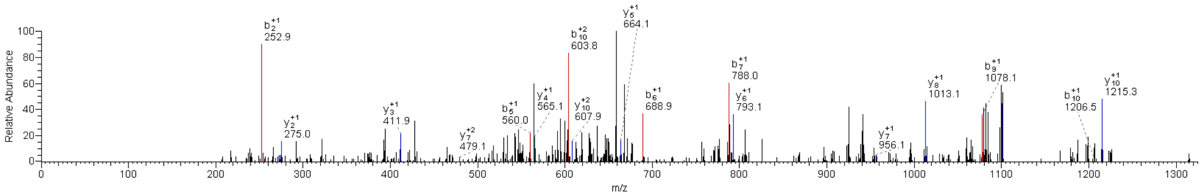

DTA for scan: 108 Precursor ion: 451.47 Charge: 3 Activation type: CID Mass type: mono
Mod's: (DAEFRHSGYVQK* -45.02150) (DAEFRHSGYVQK# -89.05000) (DAEFRHSGYVQK@ +15.99490)

	AA	A	B	B*	Bo	C	Y	Y*	Yo	Z	
1	H		138.07				-				11
2	D		253.09				1215.54				10
3	S		340.13				1100.51				9
4	G		397.15				1013.48				8
5	Y		560.21				956.46				7
6	E		689.25				793.40				6
7	V		788.32				664.35				5
8	H		925.38				565.28				4
9	H@		1078.43				428.23				3
10	Q		1206.49				275.17				2
11	K		-				147.11				1

z=1 z=2 z=3

DTA for scan: 108 Precursor ion: 451.47 Charge: 3 Activation type: CID Mass type: mono
Mod's: (DAEFRHSGYVQK* -45.02150) (DAEFRHSGYVQK# -89.05000) (DAEFRHSGYVQK@ +15.99490)

	AA	A	B	B*	Bo	C	Y	Y*	Yo	Z	
1	H		69.54				-				11
2	D		127.05				608.27				10
3	S		170.57				550.76				9
4	G		199.08				507.24				8
5	Y		280.61				478.73				7
6	E		345.13				397.20				6
7	V		394.66				332.68				5
8	H		463.19				283.15				4
9	H@		539.72				214.62				3
10	Q		603.75				138.09				2
11	K		-				74.06				1

z=1 z=2 z=3

DTA for scan: 108 Precursor ion: 451.47 Charge: 3 Activation type: CID Mass type: mono
Mod's: (DAEFRHSGYVQK* -45.02150) (DAEFRHSGYVQK# -89.05000) (DAEFRHSGYVQK@ +15.99490)

	AA	A	B	B*	Bo	C	Y	Y*	Yo	Z	
1	H		46.69				-				11
2	D		85.04				405.85				10
3	S		114.05				367.51				9
4	G		133.05				338.50				8
5	Y		187.41				319.49				7
6	E		230.42				265.14				6
7	V		263.45				222.12				5
8	H		309.13				189.10				4
9	H@		360.15				143.41				3
10	Q		402.84				92.40				2
11	K		-				49.71				1

z=1 z=2 z=3

#108-108 RT:4.89-4.89 NL: 9.86E4

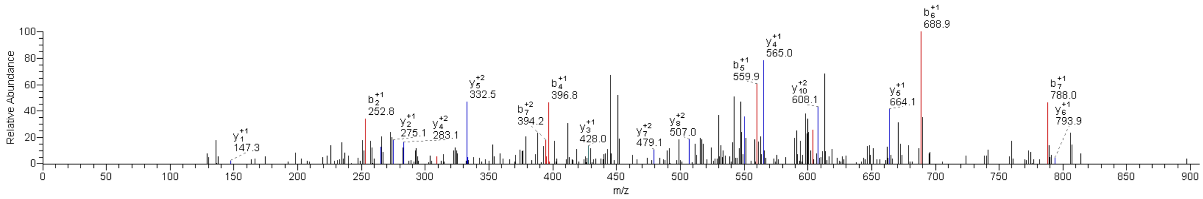

DTA for scans: 411-416 Precursor ion: 671.21 Charge: 2 Activation type: CID Mass type: mono
Mod's: (DAEFRHSGYVQK* -45.02150) (DAEFRHSGYVQK# -89.05000) (DAEFRHSGYVQK@ +15.99490)

	AA	A	B	B*	Bo	C	Y	Y*	Yo	Z	
1	L		114.09				-				12
2	V		213.16				1228.58				11
3	F@		376.22				1129.52				10
4	F		523.29				966.45				9
5	A		594.33				819.38				8
6	E		723.37				748.35				7
7	D		838.40				619.30				6
8	V		937.47				504.28				5
9	G		994.49				405.21				4
10	S		1081.52				348.19				3
11	N		1195.56				261.16				2
12	K		-				147.11				1

z=1 z=2

DTA for scans: 411-416 Precursor ion: 671.21 Charge: 2 Activation type: CID Mass type: mono
Mod's: (DAEFRHSGYVQK* -45.02150) (DAEFRHSGYVQK# -89.05000) (DAEFRHSGYVQK@ +15.99490)

	AA	A	B	B*	Bo	C	Y	Y*	Yo	Z	
1	L		57.55				-				12
2	V		107.08				614.80				11
3	F@		188.62				565.26				10
4	F		262.15				483.73				9
5	A		297.67				410.20				8
6	E		362.19				374.68				7
7	D		419.70				310.16				6
8	V		469.24				252.64				5
9	G		497.75				203.11				4
10	S		541.26				174.60				3
11	N		598.29				131.08				2
12	K		-				74.06				1

z=1 z=2

#411-416 RT:13.05-13.17 NL: 2.19E6

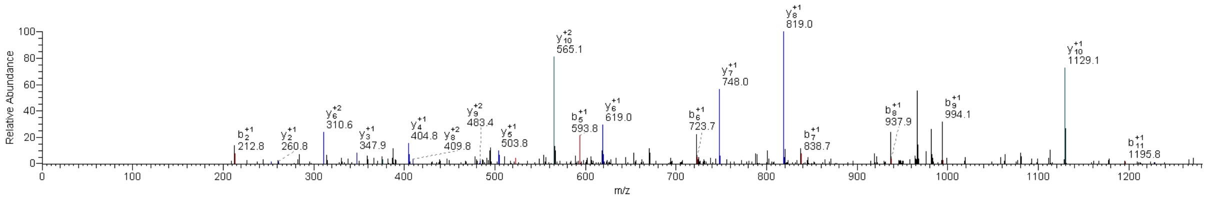

DTA for scans: 411-416 Precursor ion: 671.21 Charge: 2 Activation type: CID Mass type: mono
Mod's: (DAEFRHSGYVQK* -45.02150) (DAEFRHSGYVQK# -89.05000) (DAEFRHSGYVQK@ +15.99490)

	AA	A	B	B*	Bo	C	Y	Y*	Yo	Z	
1	L		114.09				-				12
2	V		213.16				1228.58				11
3	F		360.23				1129.52				10
4	F@		523.29				982.45				9
5	A		594.33				819.38				8
6	E		723.37				748.35				7
7	D		838.40				619.30				6
8	V		937.47				504.28				5
9	G		994.49				405.21				4
10	S		1081.52				348.19				3
11	N		1195.56				261.16				2
12	K		-				147.11				1

z=1 z=2

DTA for scans: 411-416 Precursor ion: 671.21 Charge: 2 Activation type: CID Mass type: mono
Mod's: (DAEFRHSGYVQK* -45.02150) (DAEFRHSGYVQK# -89.05000) (DAEFRHSGYVQK@ +15.99490)

	AA	A	B	B*	Bo	C	Y	Y*	Yo	Z	
1	L		57.55				-				12
2	V		107.08				614.80				11
3	F		180.62				565.26				10
4	F@		262.15				491.73				9
5	A		297.67				410.20				8
6	E		362.19				374.68				7
7	D		419.70				310.16				6
8	V		469.24				252.64				5
9	G		497.75				203.11				4
10	S		541.26				174.60				3
11	N		598.29				131.08				2
12	K		-				74.06				1

z=1 z=2

#411-416 RT:13.05-13.17 NL: 2.19E6

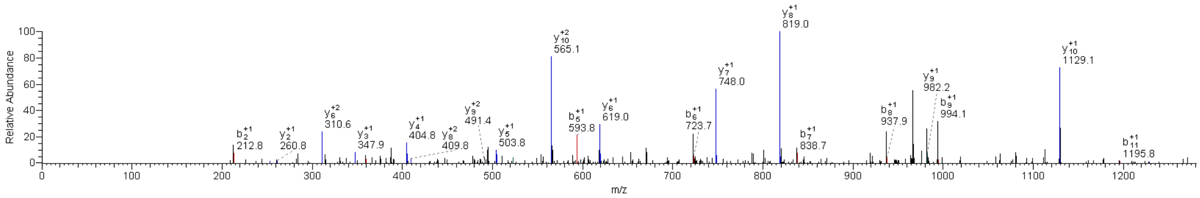

DTA for scan: 923 Precursor ion: 551.32 Charge: 2 Activation type: CID Mass type: mono
Mod's: (DAEFRHSGYVQKAIM* -89.04768) (DAEFRHSGYVQKAIM# -45.02146) (DAEFRHSGYVQKAIM@ +15.99492) (DAEFRHSGYVQKAIM^ +13.97927)

	AA	A	B	B*	Bo	C	Y	Y*	Yo	Z	
1	G		58.03				-				12
2	A		129.07				1044.61				11
3	I		242.15				973.58				10
4	I		355.23				860.49				9
5	G		412.26				747.41				8
6	L		525.34				690.39				7
7	M@		672.37				577.30				6
8	V		771.44				430.27				5
9	G		828.46				331.20				4
10	G		885.49				274.18				3
11	V		984.55				217.15				2
12	V		-				118.09				1

z=1 z=2

#923-923 RT:9.59-9.59 NL: 8.81E4

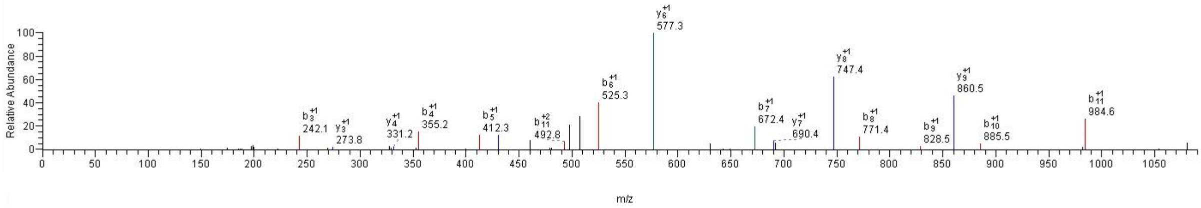

Figure S2. Analysis of $^{ox}A\beta_{1\text{-}40}$. Series of b and y ions (charge 1^+, 2^+ and 3^+) used for the identification of the oxidation of His13, His14, Phe19, Phe20, and Met35, and respective corresponding CID spectra of HDSGYEVHHQK+16 (doubly and triply protonated, precursor ions at m/z 676.94 and m/z 451.47), LVFFAEDVGSNK+16 (doubly protonated, precursor ion at m/z 671.21) and GAIIGLMVGGVV+16 (doubly protonated, precursor ion at m/z 551.32).

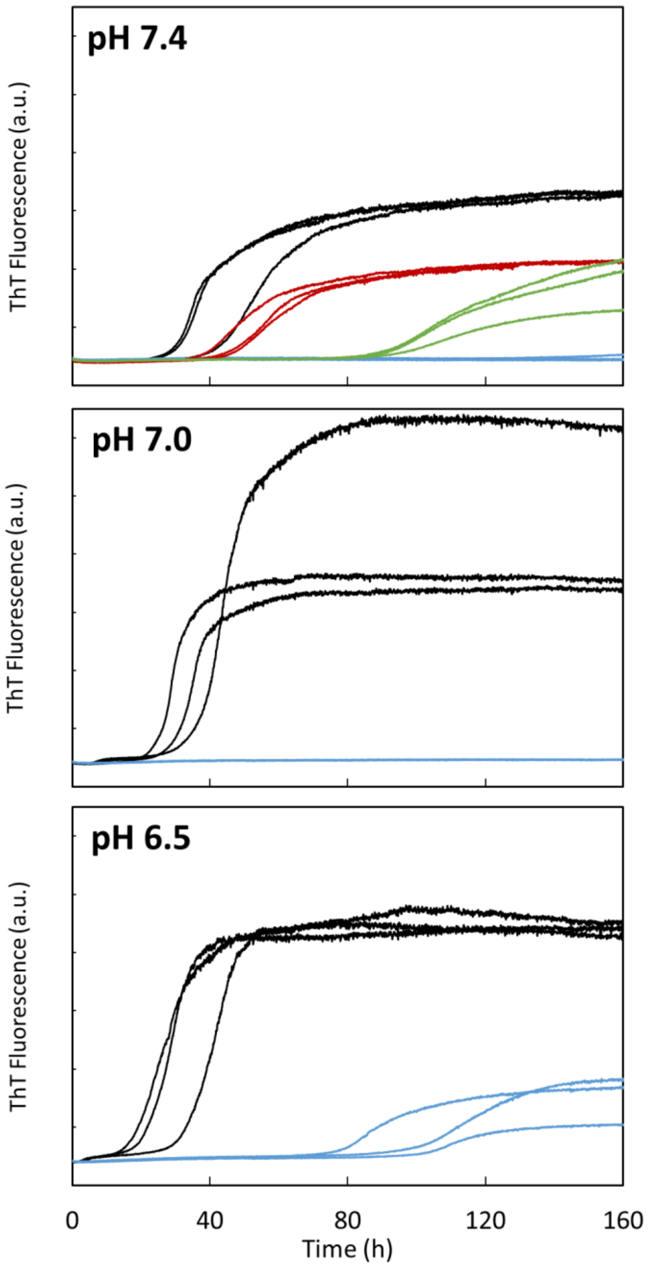

Figure S3. Kinetic monitoring of $A\beta_{1-40}$ and ${}^{ox}A\beta_{1-40}$ co-assembly using ThT fluorescence as a function of pH. Curves are shown as triplicate. $[A\beta_{1-40}]$ = 20µM (black); $[{}^{ox}A\beta_{1-40}]$ = 20µM (blue); $[A\beta_{1-40}]$ = $[{}^{ox}A\beta_{1-40}]$ = 10µM (green) and $[A\beta_{1-40}]$ = 10µM (red). pH 7.4 (panel A), 7.0 (panel B), 6.5 (panel C). Data from experiment N°3. HEPES buffer 50 mM with [NaCl] = 65 mM. Y-axis corresponds to ThT Fluorescence in arbitrary unit (a.u.); data are directly comparable between them (same y-scale).

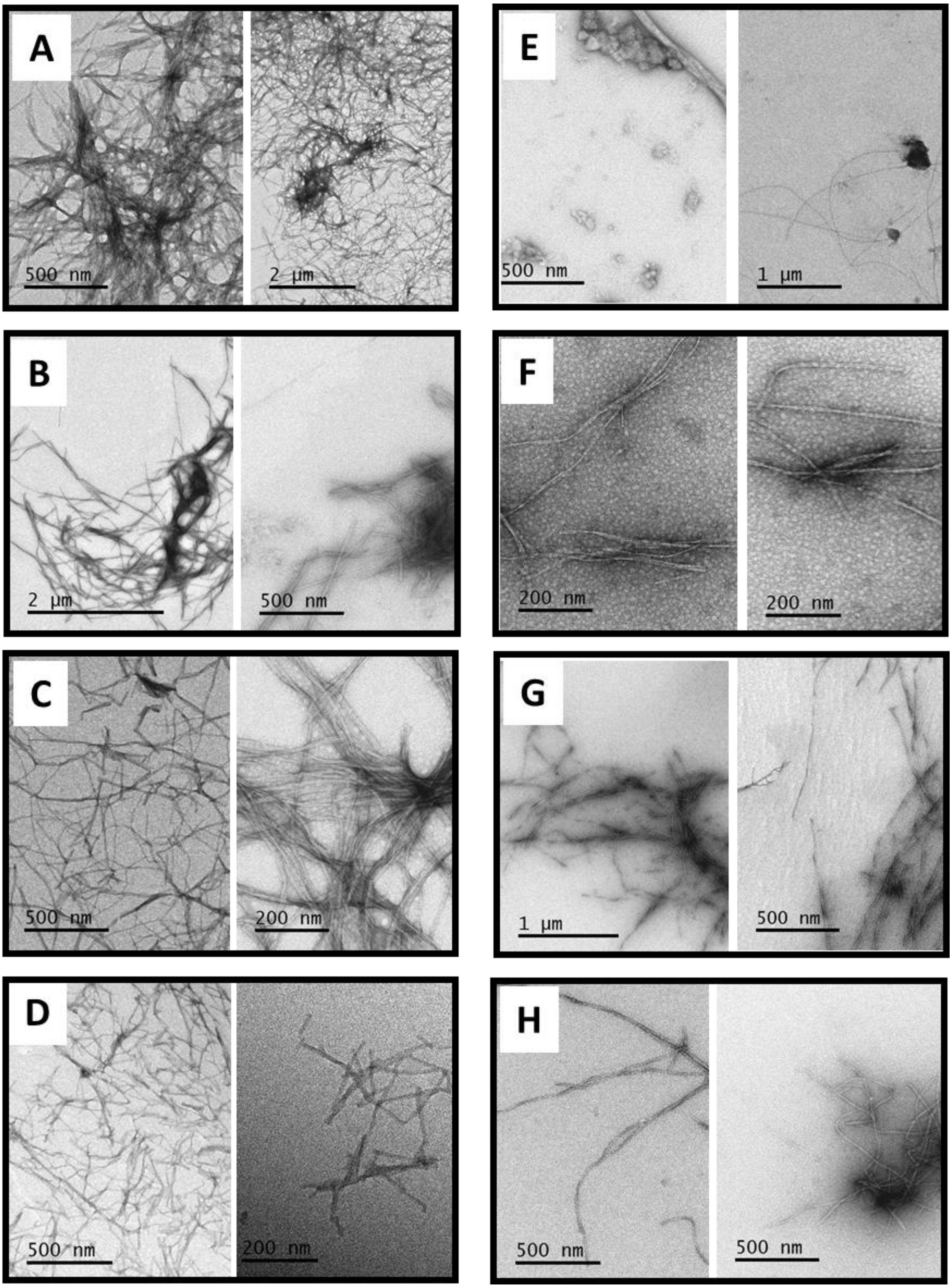

Figure S4. Selected TEM pictures of $A\beta_{1-40}$ (left) and $^{ox}A\beta_{1-40}$ (right) at 20 µM, at pH 7.4 (panels A and E), 7.0 (panels B and F), at pH 6.5 (C and G) and 6.0 (panels (D and G) taken at the end of the ThT fluorescence experiment N°1, Hepes buffer 50 mM, [NaCl] = 65 mM. Two shots are given to better illustrate the heterogeneity of the assemblies formed.

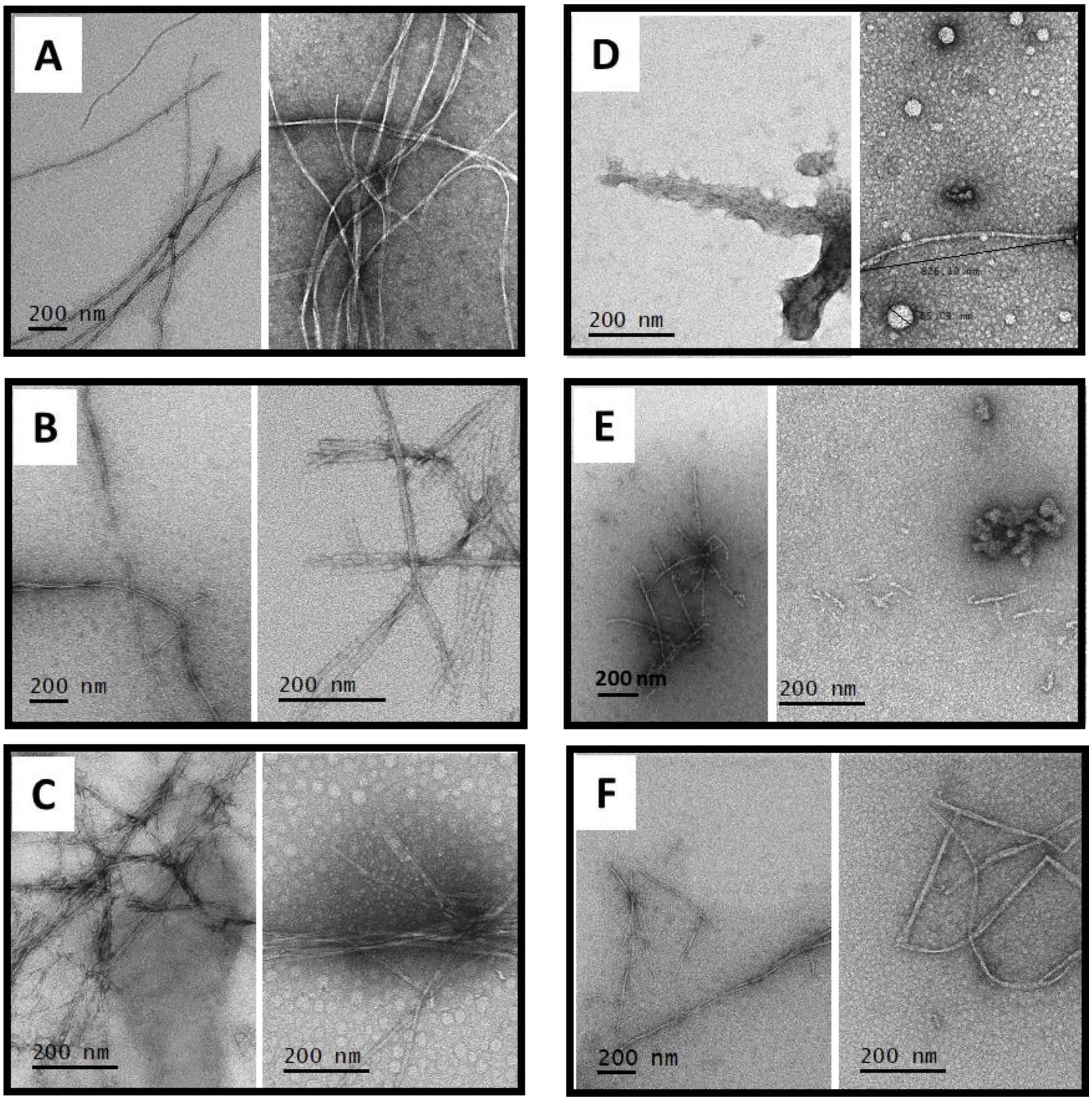

Figure S5. Selected TEM pictures of A$\beta_{1\text{-}40}$ (left) and ^{ox}A$\beta_{1\text{-}40}$ (right) at 20 µM, at pH 7.4 (panels A and D), 7.0 (panels B and E), at pH 6.5 (C and F) taken at the end of the ThT fluorescence experiment N°3, Hepes buffer 50 mM, [NaCl] = 65 mM. Two shots are given to better illustrate the heterogeneity of the assemblies formed.

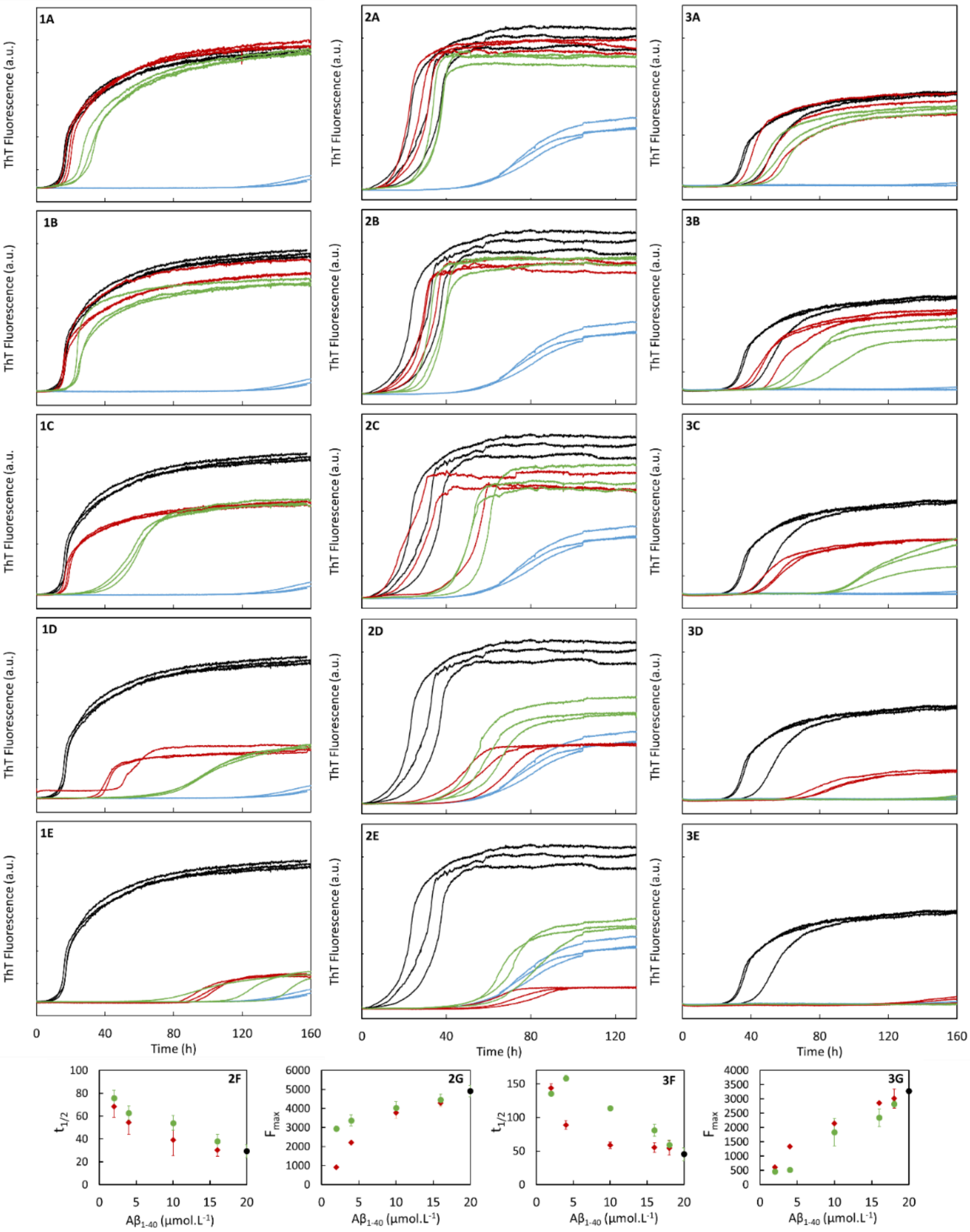

Figure S6. Kinetic monitoring of $A\beta_{1\text{-}40}$ and $^{ox}A\beta_{1\text{-}40}$ co-assembly using ThT fluorescence as a function of ratio between peptides in three independent experiments (Experiments N°1 (left, pH 7.0), 2 (middle, pH 7.0) and 3 (right, pH 7.4)). Curves are shown as triplicate. $[A\beta_{1\text{-}40}]$ = 20µM (black); $[^{ox}A\beta_{1\text{-}40}]$ = 20µM (blue); $[A\beta_{1\text{-}40}]$ = 18µM & $[^{ox}A\beta_{1\text{-}40}]$ = 2µM (green) ; $[A\beta_{1\text{-}40}]$ = 18µM (red) (panels 1A, 2A, 3A) ; $[A\beta_{1\text{-}40}]$ = 16µM & $[^{ox}A\beta_{1\text{-}40}]$ = 4µM (green) ; $[A\beta_{1\text{-}40}]$ = 16µM (red) (panels 1B, 2B, 2C), $[A\beta_{1\text{-}40}]$ = $[^{ox}A\beta_{1\text{-}40}]$ = 10µM (green) ; $[A\beta_{1\text{-}40}]$ = 10µM (red) (panels 1C, 2C, 3C) ; $[A\beta_{1\text{-}40}]$ = 4µM & $[^{ox}A\beta_{1\text{-}40}]$ = 16µM (green) ; $[A\beta_{1\text{-}40}]$ = 4µM (red) (panels 1D, 2D, 2D) ; $[A\beta_{1\text{-}40}]$ = 2µM & $[^{ox}A\beta_{1\text{-}40}]$ = 18µM (green) ; $[A\beta_{1\text{-}40}]$ = 18µM (red) (panels 1E, 2E, 3E). Data are directly comparable between them (same y-scale).Panels F and G: Average $t_{1/2}$ and F_{max}, values as a function of the $A\beta_{1\text{-}40}$ concentration with $^{ox}A\beta_{1\text{-}40}$ present ($[A\beta_{1\text{-}40}]$ + $[^{ox}A\beta_{1\text{-}40}]$ = 20 µM) (green dots) or without (red dots) for experiments N°2 and 3. Note that for experiment N°2, the real concentration of $A\beta_{1\text{-}40}$ is higher than the theoretical one given in the x-axis due to non-negligible contribution for non-oxidized peptide in the $^{ox}A\beta_{1\text{-}40}$ sample. This explain why the delaying effect of $^{ox}A\beta_{1\text{-}40}$ is not as obvious as for experiments N°1 (full text) and 3 and why the F_{max} value is higher at low $[A\beta_{1\text{-}40}]$ than in experiment N°1 (full text) and 3.

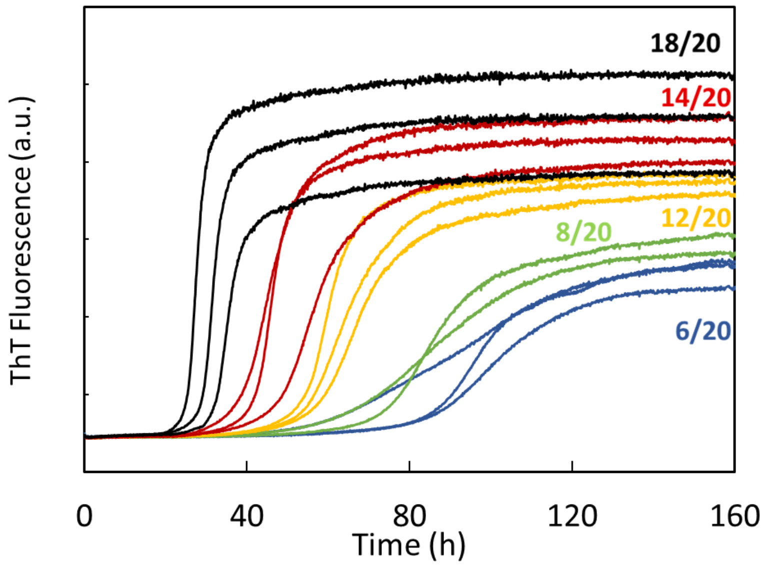

Figure S7. Kinetic monitoring of A$\beta_{1\text{-}40}$ and ^{ox}A$\beta_{1\text{-}40}$ co-assembly using ThT fluorescence as a function of addition of A$\beta_{1\text{-}40}$ to [A$\beta_{1\text{-}40}$]=20µM. Curves are shown as triplicate. All curves [^{ox}A$\beta_{1\text{-}40}$] = 20 µM + [A$\beta_{1\text{-}40}$] = 6 µM (dark blue), 8 µM (green), 12 µM (yellow), 14µM (red) , 18 µM (black). From experiment N°1. HEPES buffer 50 mM, [NaCl] = 65 mM, pH 7.4.

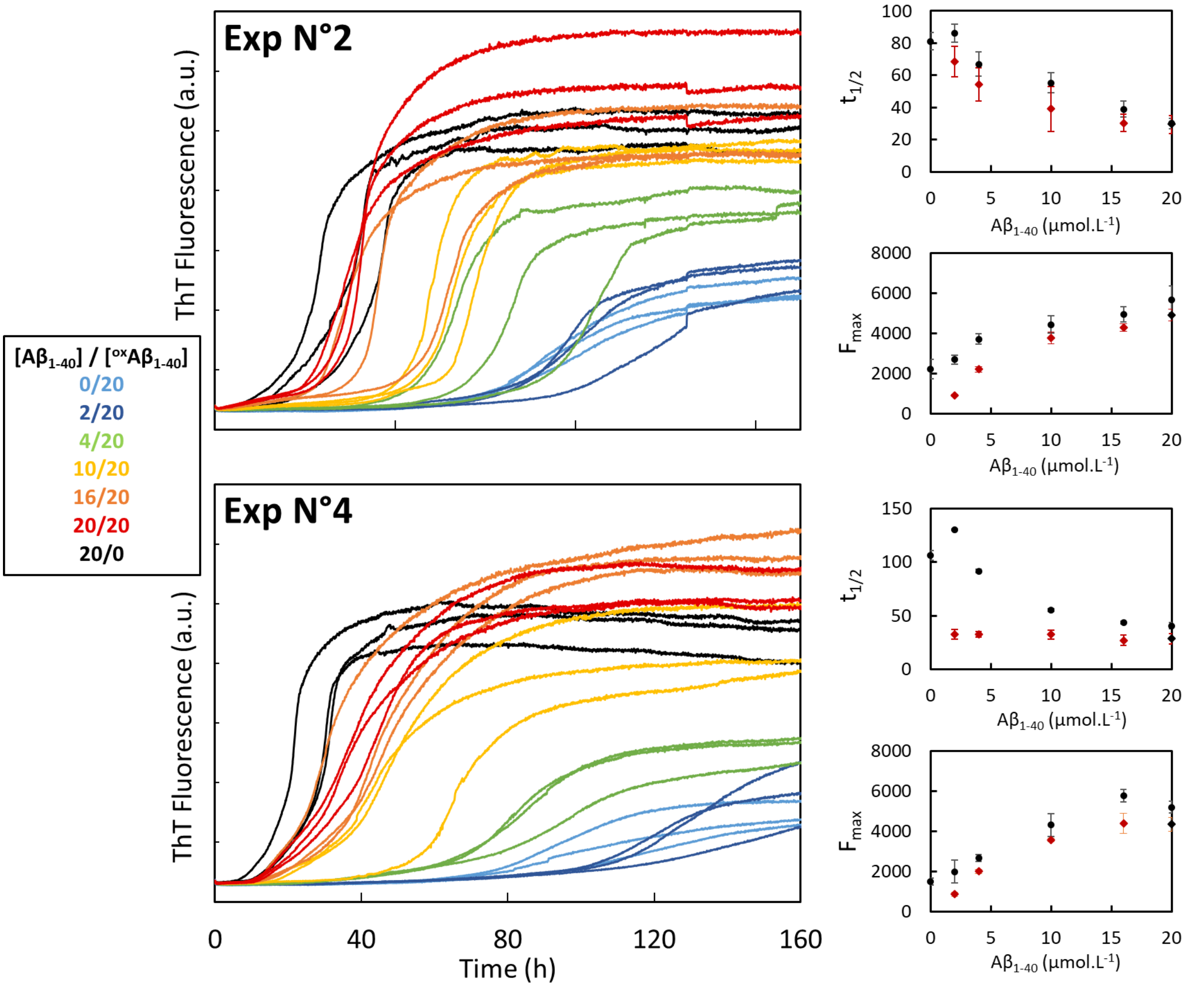

Figure S8. Kinetic monitoring of $A\beta_{1\text{-}40}$ and ${}^{ox}A\beta_{1\text{-}40}$ co-assembly using ThT fluorescence as a function of addition of $A\beta_{1\text{-}40}$ to ${}^{ox}A\beta_{1\text{-}40}$. Curves are shown as triplicate. [$A\beta_{1\text{-}40}$] = 20µM (black); [${}^{ox}A\beta_{1\text{-}40}$] = 20µM (blue); [Aβox] = 20µM + [$A\beta_{1\text{-}40}$] = 2µM (dark blue) ; [${}^{ox}A\beta_{1\text{-}40}$] = 20µM + [$A\beta_{1\text{-}40}$] = 4µM (green) ; [${}^{ox}A\beta_{1\text{-}40}$] = 20µM + [$A\beta_{1\text{-}40}$] = 10µM (yellow) ; [${}^{ox}A\beta_{1\text{-}40}$] = 20µM + [$A\beta_{1\text{-}40}$] = 16µM (orange) ; [${}^{ox}A\beta_{1\text{-}40}$] = 20µM + [$A\beta_{1\text{-}40}$] = 20µM (red) (left panel) and average F_{max}, and $t_{1/2}$ values as a function of the $A\beta_{1\text{-}40}$ added for 20µM ${}^{ox}A\beta_{1\text{-}40}$ (black dots) and reference without ${}^{ox}A\beta_{1\text{-}40}$ (dark red dots) (right panel). HEPES buffer 50 mM with NaCl 65 mM, pH 7.0. Y-axis corresponds to ThT Fluorescence in arbitrary unit (a.u.); data are directly comparable between them (same y-scale). Note that for experiment N°2, the real concentration of $A\beta_{1\text{-}40}$ is higher than the theoretical one given in the x-axis due to non-negligible contribution for non-oxidized peptide in the ${}^{ox}A\beta_{1\text{-}40}$ sample. This explain why the F_{max} value is higher at low [$A\beta_{1\text{-}40}$] than in experiment N°1 (full text) and 4.

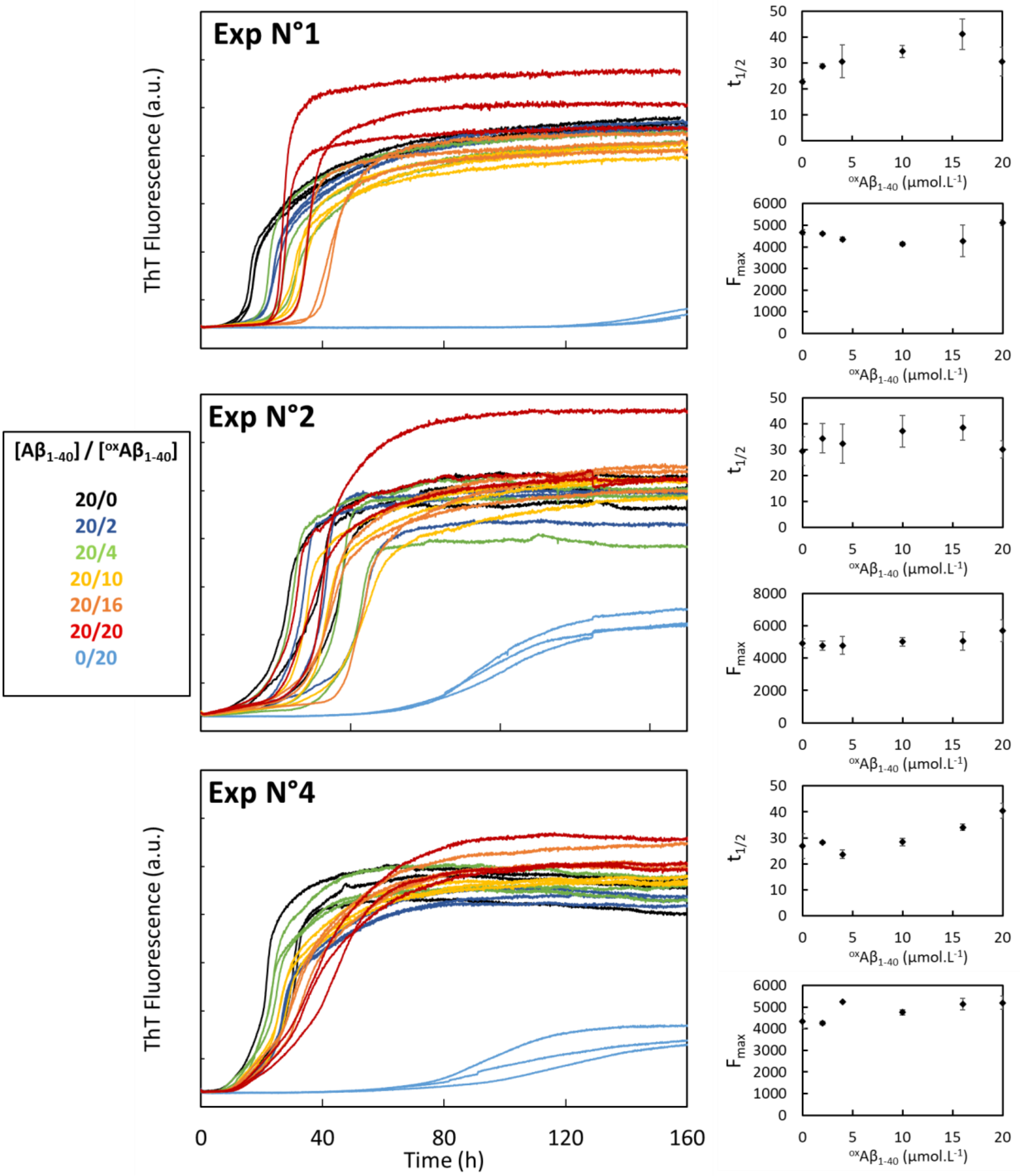

Figure S9. Kinetic monitoring of $A\beta_{1\text{-}40}$ and ${}^{ox}A\beta_{1\text{-}40}$ co-assembly using ThT fluorescence as a function of addition of ${}^{ox}A\beta_{1\text{-}40}$ to $A\beta_{1\text{-}40}$. Curves are shown as triplicate. [$A\beta_{1\text{-}40}$] = 20µM (black); [${}^{ox}A\beta_{1\text{-}40}$] = 20µM (blue); [$A\beta_{1\text{-}40}$] = 20µM + [${}^{ox}A\beta_{1\text{-}40}$] = 2µM (dark blue) ; [$A\beta_{1\text{-}40}$] = 20µM + [${}^{ox}A\beta_{1\text{-}40}$] = 4µM (green) ; [$A\beta_{1\text{-}40}$] = 20µM + [${}^{ox}A\beta_{1\text{-}40}$] = 10µM (yellow) ; [$A\beta_{1\text{-}40}$] = 20µM + [${}^{ox}A\beta_{1\text{-}40}$] = 16µM (orange) ; [$A\beta_{1\text{-}40}$] = 20µM + [${}^{ox}A\beta_{1\text{-}40}$] = 20µM (red) (left panel) and average F_{max}, and $t_{1/2}$ values as a function of the ${}^{ox}A\beta_{1\text{-}40}$ added (right panel). From experiments N°1, 2 and 4. HEPES buffer 50 mM with NaCl 65 mM, pH 7.4 (Exp N°1) or pH 7.0 (Exp N°2 and 4).

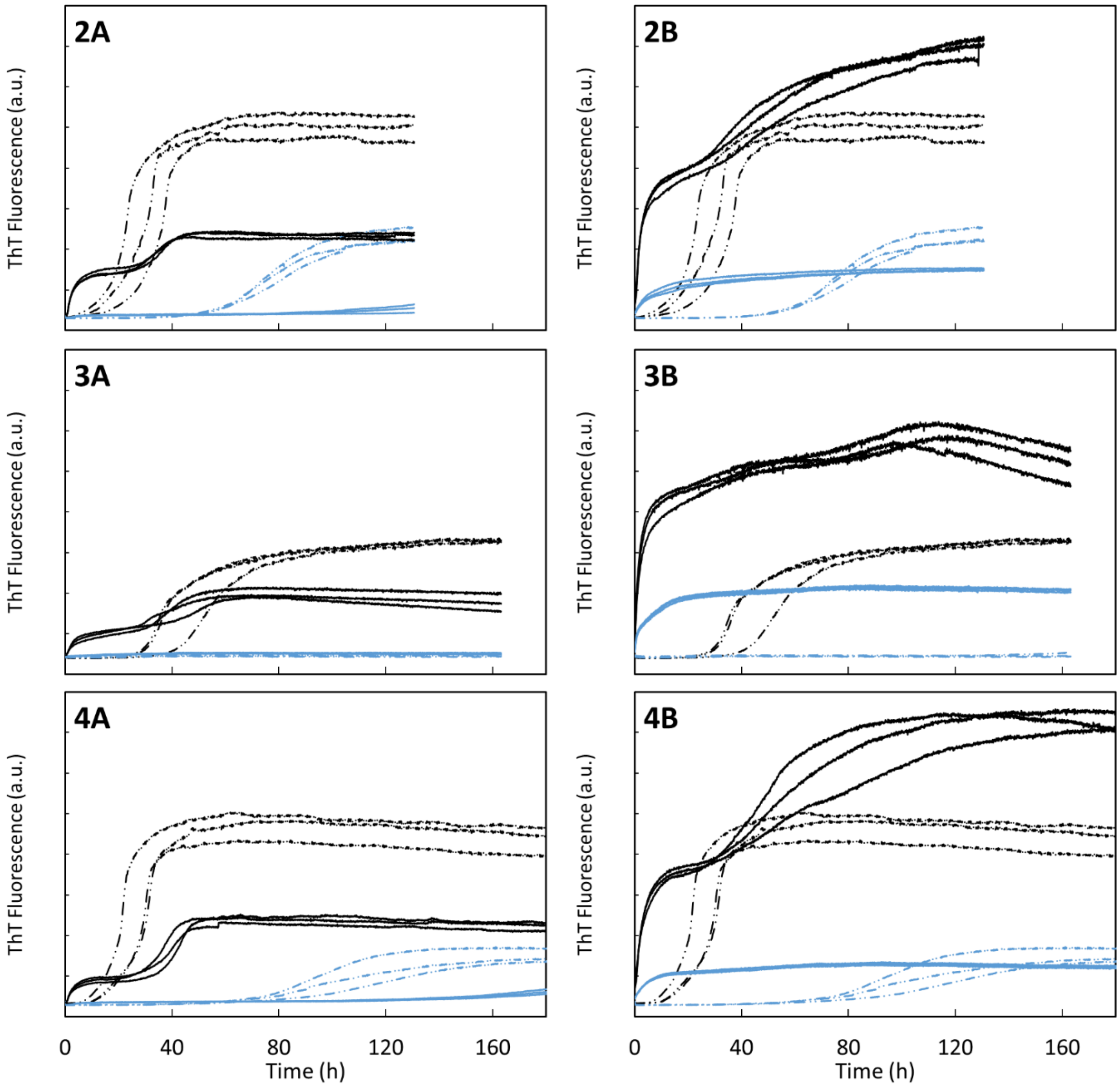

Figure S10. Kinetic monitoring of Aβ_{1-40} and ^{ox}Aβ_{1-40} co-assembly in presence of Cu(II) or Zn(II) using ThT fluorescence for 3 independent experiments at two different pH values. Curves are shown as triplicate. [Aβ_{1-40}] = 20µM (black); [^{ox}Aβ_{1-40}] = 20µM (blue); Apo peptides (dashed curves), with Cu(II) (18 µM, Panel A, solid curves) or with Zn(II) (18 µM, Panel B, solid curves) at pH 7.0 (Exp. N°2 and 4) or pH 7.4 (Exp N° 3). HEPES buffer 50 mM with [NaCl] = 65 mM.

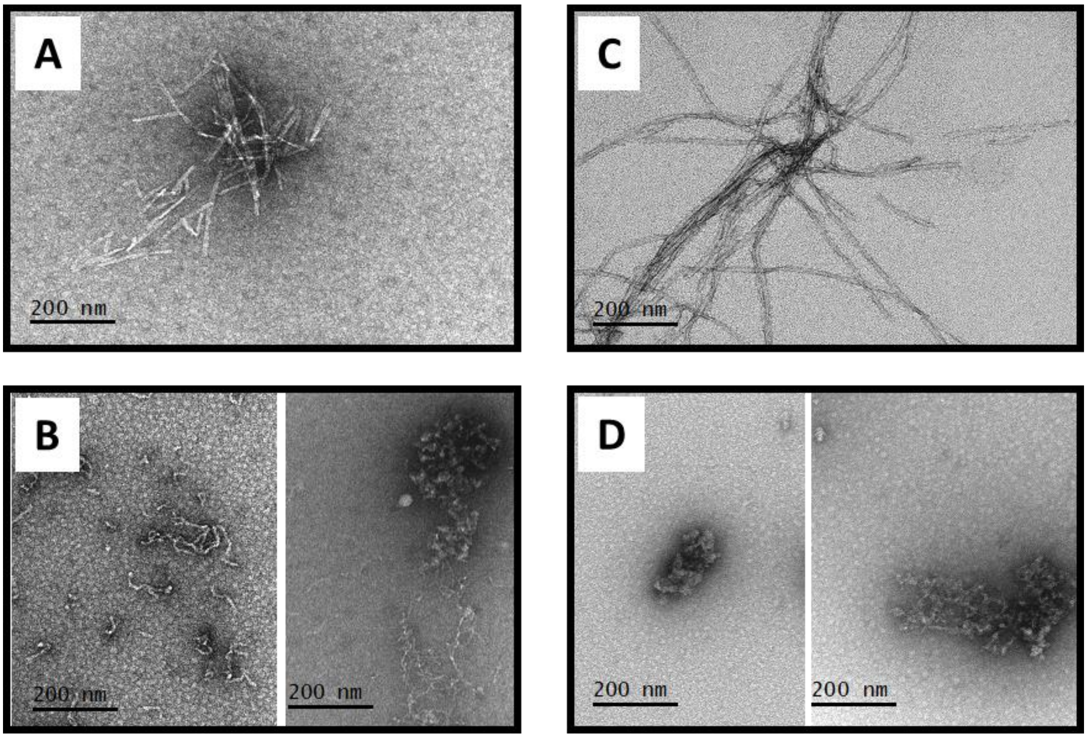

Figure S11. Selected TEM pictures of Cu(Aβ_{1-40}) and Cu(^{ox}Aβ_{1-40}) at 20 µM (panels A and B, respectively); and of Zn(Aβ_{1-40}) and Zn(^{ox}Aβ_{1-40}) at 20 µM (panels C and D, respectively), respectively), taken at the end of the ThT Fluorescence experiment N°3 (Hepes buffer 50 mM, [NaCl] = 65 mM, pH 7.4). When relevant, two shots are given to better illustrate the heterogeneity of the assemblies formed.

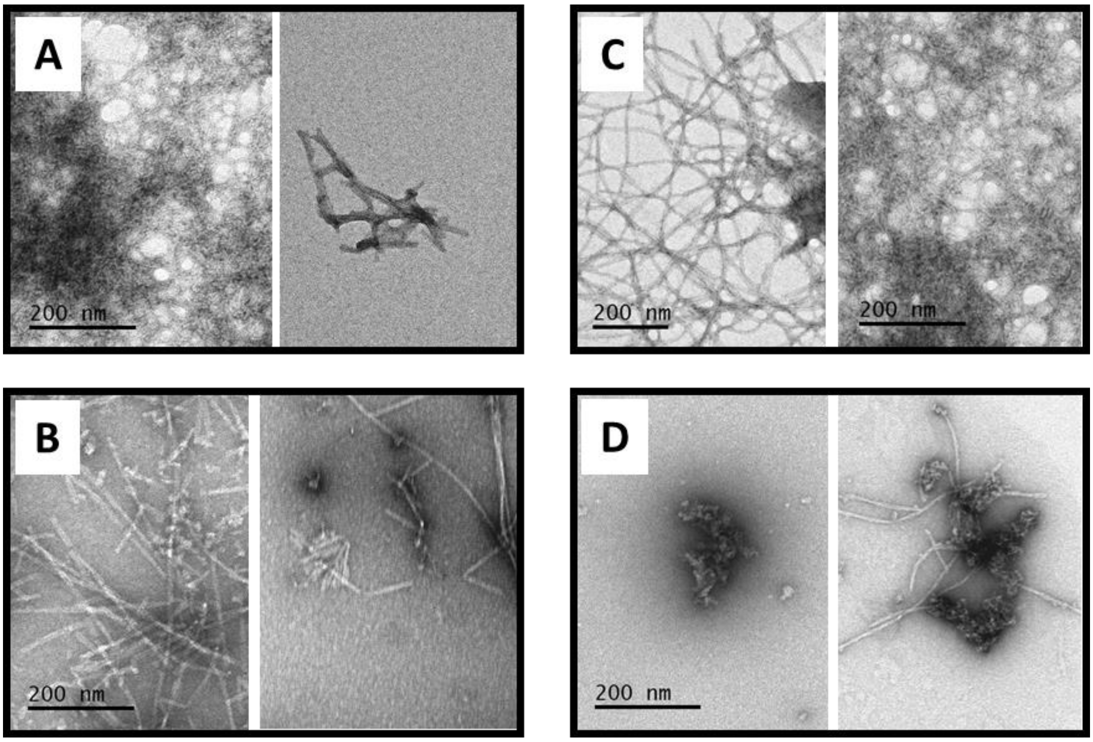

Figure S12. Selected TEM pictures of Cu(Aβ_{1-40}) and Cu(^{ox}Aβ_{1-40}) at 20 µM (panels A and B, respectively); and of Zn(Aβ_{1-40}) and Zn(^{ox}Aβ_{1-40}) at 20 µM (panels C and D, respectively), respectively), taken at the end of the ThT Fluorescence experiment N°4 (Hepes buffer 50 mM, [NaCl] = 65 mM, pH 7.0). Two shots are given to better illustrate the heterogeneity of the assemblies formed.
